# Dietary *Aronia melanocarpa* extract enhances mTORC1 signaling, but has no effect on protein synthesis and protein breakdown-related signaling, in response to resistance exercise in rat skeletal muscle

**DOI:** 10.1186/s12970-019-0328-1

**Published:** 2019-12-11

**Authors:** Yuhei Makanae, Satoru Ato, Kohei Kido, Satoshi Fujita

**Affiliations:** 1 Department of Physical Education, National Defense Academy, Yokosuka, Kanagawa Japan; 2 Ritsumeikan Global Innovation Research Organization, Ritsumeikan University, Kusatsu, Shiga Japan; 3 Faculty of Sports and Health Science, Ritsumeikan University, Kusatsu, Shiga Japan; 4 Department of Life Science and Applied Chemistry, Nagoya Institute of Technology, Nagoya, Japan; 5 Laboratory of Sports and Exercise Medicine, Graduate School of Human and Envionmental Studies, Kyoto University, Kyoto, Japan

**Keywords:** *Aronia melanocarpa*, mTORC1, Resistance exercise, Skeletal muscle, Ursolic acid

## Abstract

**Background:**

Ursolic acid altered muscle protein metabolism in normal and resting conditions after acute resistance exercise, suggesting that eating fruits rich in ursolic acid could enhance muscle protein synthesis and decrease muscle degradation. *Aronia melanocarpa*, a member of the family Rosaceae and native to North America and Eastern Canada, is rich in ursolic acid. In this study, we examined the effects of *A. melanocarpa* extract (AME) supplementation on the mTORC1 signaling pathway and muscle degradation-related factors in rats, both alone and in combination with resistance exercise.

**Methods:**

Male Sprague-Dawley rats were divided into AME and normal chow (NOR) groups. AME group was fed chow providing a dose of 3 g/kg of AME and 115 mg/kg of ursolic acid for 7 days, whereas NOR rats were fed normal powder chow. The right gastrocnemius muscle of each animal was isometrically exercised (5 sets of ten 3-s contractions, with a 7-s interval between contractions and 3-min rest intervals between sets), while the left gastrocnemius muscle served as an internal control. Western blotting and real-time polymerase chain reaction were used to assess expression of factors involved in the mTORC1 signaling pathway and muscle degradation.

**Results:**

At 1 h after resistance exercise, phosphorylation of ERK1/2 was significantly increased by AME consumption. At 6 h after resistance exercise, AME consumption significantly increased the phosphorylation of Akt, p70S6K, rpS6, and AMPK. It also increased MAFbx expression. Furthermore, AME significantly increased the phosphorylation of p70S6K and rpS6 in response to resistance exercise. However, AME did not increase muscle protein synthesis (MPS) after resistance exercise. AME did not affect the expression of any of the mediators of protein degradation, with the exception of MAFbx.

**Conclusions:**

Dietary AME enhanced mTORC1 activation in response to resistance exercise without increasing MPS. Moreover, it neither accelerated muscle protein degradation nor otherwise negatively affected protein metabolism. Further study is needed to clarify the effect of the combination of AME and chronic resistance training on muscle hypertrophy.

## Background

Maintenance of skeletal muscle mass, which is required for motion and is responsible for more energy consumption than any other tissue in the body [1], is important for promoting health and quality of life. Muscle mass is determined by the net balance of protein synthesis and protein breakdown. Previous studies have demonstrated that mechanistic target of rapamycin complex 1 (mTORC1) and muscle protein synthesis (MPS) are key positive regulators of skeletal muscle mass [2, 3]. The p70S6 kinase (p70S6K) and ribosomal protein S6 (rpS6) are downstream substrates of mTORC1. In particular, p70S6K phosphorylation has been correlated with the magnitude of muscle hypertrophy [4, 5]. Our previous study has demonstrated that administration of rapamycin, an mTORC1 inhibitor, inhibited p70S6K phosphorylation and attenuated muscle hypertrophy in response to resistance training [6]. Thus, p70S6K is a potential marker for resistance training-induced muscle hypertrophy, although other signaling substrates also contribute to muscle MPS and muscle hypertrophy [6, 7]. In contrast, AMP-activated protein kinase (AMPK) acts as a cellular energy sensor and regulates mediators of muscle protein degradation, including the ubiquitin-proteasome system (UPS) and the autophagy-lysosomal system [8, 9]. In UPS-mediated protein degradation, two muscle-specific ubiquitin ligases polyubiquitinate target proteins leading to degradation via proteasomes [10]. The autophagy-lysosomal system is another major protein degradation pathway and is regulated by UNC-51-like kinase 1 (ULK1) [11].

Numerous reports have demonstrated that resistance exercise and nutrients regulate muscle protein synthesis and breakdown [4, 6, 12–16]. One nutrient in particular, ursolic acid, a lipophilic pentacyclic triterpenoid, alters muscle metabolism [14, 15]. Kunkel et al. demonstrated that ursolic acid activates mTORC1 signaling and decreases mRNA expression of muscle atrophy F box (MAFbx) and muscle-specific RING finger 1 (MuRF1) in skeletal muscle [14]. In addition, these researchers demonstrated that supplementation with ursolic acid induced muscle hypertrophy and inhibited denervation-induced muscle atrophy [14].

*Aronia melanocarpa*, a member of the family Rosaceae and native to North America and Eastern Canada, is rich in ursolic acid. The fruit of this plant has several activities in common with ursolic acid [17–19]. Thus, the consumption of fruits with high ursolic acid content could enhance muscle protein synthesis and decrease muscle degradation. However, no study has investigated the effect of *A. melanocarpa* on muscle protein metabolism.

An acute bout of resistance exercise increases mTORC1 activity and rates of protein synthesis/breakdown, causing skeletal muscle hypertrophy [4, 6, 12, 16]. Several studies have shown that nutritional supplementation, including with amino acids and protein, enhances these increases in mTORC1 activity [20–22] and reduces protein breakdown [23], resulting in acceleration of muscle hypertrophy [24]. Our group has demonstrated that acute ursolic acid injection augmented the resistance exercise-induced mTORC1 response [15]. A recent study demonstrated that mTORC1 activation is necessary for muscle hypertrophy induced by mechanical load [25]. Furthermore, Mitchell et al. reported a correlation between mTORC1 activity and resistance training-induced muscle hypertrophy [5]. Thus, mTORC1 may be a predictor of muscle hypertrophy. Although in our previous work, we did not measure the effect of the combination of ursolic acid supplementation and chronic resistance training [15], the findings suggested that ursolic acid supplementation may be effective to induce muscle hypertrophy. Thus, *A. melanocarpa*, with its high concentration of ursolic acid, may enhance resistance exercise-induced muscle hypertrophy via an increase in mTORC1 activity. mTORC1 is involved in the regulation of not only protein synthesis but also protein breakdown via inhibition of ubiquitin ligase expression and ULK1 kinase activity [26, 27]. Therefore, the addition of *A. melanocarpa* supplementation to exercise may further positively affect muscle metabolism in response to an acute bout of resistance exercise.

In this study, we examined the effects of supplementation with *A. melanocarpa* extract (AME) on the mTORC1 signaling pathway, MPS, and muscle degradation-related factors in rats, both alone and in combination with resistance exercise.

## Methods

### Animals

Male Sprague-Dawley rats (age 10 weeks, body weight 310–340 g) were obtained from CLEA Japan (Tokyo, Japan). All rats were housed for 1 week at 22 °C with a 12/12-h light/dark cycle and provided with commercial solid rat chow (CE2; CLEA Japan) and drinking water ad libitum. One week prior to the study, the solid chow was replaced with powder chow (CE2; CLEA Japan), which was later used for administration of AME. This study was approved by the Ethics Committee for Animal Experiments of Ritsumeikan University (BKC2018–044).

#### AME administration and experimental protocol

**Fig. 1:**
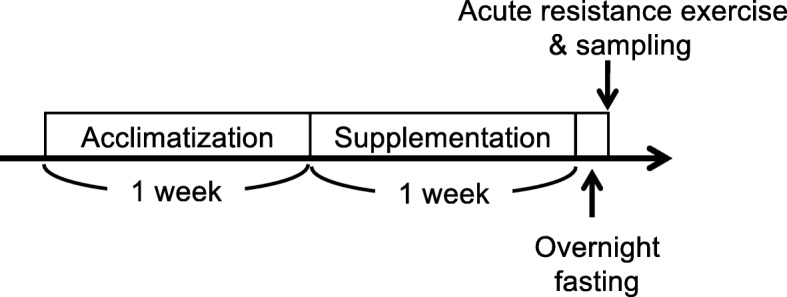
Schematic of the experimental protocol

After acclimatization for 1 week, the rats were divided into the AME and normal chow (NOR) groups. The AME rats were provided chow containing approximately 2.9 g/kg body weight of AME (Table 1), which provided approximately 115 mg/kg body weight of ursolic acid, for 7 days, while NOR rats were provided unsupplemented powder chow for 7 days. A previous study demonstrated that chow including 0.14% ursolic acid regulated muscle metabolism in mice [14], but there are differences in the body weight and amount of food consumption between rats and mice. Thus, we supplemented the chow with a concentration of AME that contained the same amount of ursolic acid as in the previous study. The components of AME and their relative amounts are shown in Table 1. The amount of food consumed and body weight were measured at day 2, 4, and 7 of the AME supplementation period. At 7 days, the right gastrocnemius muscle was isometrically exercised after 12 h of fasting overnight (Fig. 1). Under anesthesia, rats were euthanized by exsanguination at 1 and 6 h after completion of the resistance exercise, followed by the removal of the gastrocnemius muscles of both legs (*n* = 5 for each time point). Tissues were rapidly frozen in liquid N_2_ and stored at − 80 °C until analysis.

**Table 1:** Components of AME

Component	Amount (mg/g AME chow)
Ursolic acid	2
Acetylursolic acid	1.6
Oleanolic acid	0.8
Acetyloleanolic acid	0.6
Anthocyanin	2.2
Protocatechuic acid	0.12
Chlorogenic acid	0.16
Isoquercitin	0.045
Quercetin	0.0035
β-cryptoxanthin	0.00085
β-carotene	0.00515

*AME Aronia melanocarpa* extract

#### Resistance exercise protocol

Under isoflurane anesthesia, the right lower hindlimb of each rat was shaved and cleaned with alcohol wipes. Animals were positioned with the right foot on the footplate (ankle joint at 90°) in the prone posture. The triceps surae muscle was stimulated percutaneously with 10 mm × 5 mm electrodes (Vitrode V, Ag/AgCl; Nihon Kohden, Tokyo, Japan) connected to an electric stimulator and an isolator (SS-104 J; Nihon Kohden) [28]. The right gastrocnemius muscle was isometrically exercised (5 sets of ten 3-s contractions, with a 7-s interval between contractions and 3-min rest intervals between sets), while the left gastrocnemius muscle served as a control. Voltage (~ 30 V) and stimulation frequency (100 Hz) were adjusted to produce maximal isometric tension [15].

#### Western blotting

Western blotting was performed as previously reported [29]. Briefly, muscle samples were crushed while frozen and homogenized in homogenization buffer containing 20 mM Tris-HCl (pH 7.5), 1 mM Na_2_EDTA, 1% NP-40, 2.5 mM sodium pyrophosphate, 1% sodium deoxycholate, 1 mM EGTA, 150 mM NaCl, 1 mM β-glycerophosphate, 1 mM Na_3_VO_4_, 1 g/ml leupeptin, and protease and phosphatase inhibitor cocktail (Thermo Fisher Scientific, Waltham, MA, USA). Homogenates were centrifuged at 10,000×g for 10 min at 4 °C. After the supernatant was removed, the protein concentration was determined using the Protein Assay Rapid kit (WAKO, Osaka, Japan). Samples were diluted in 3× sample buffer containing 15% v/v β-mercaptoethanol, 6% w/v sodium dodecyl sulfate (SDS), 187.5 mM Tris-HCl (pH 6.8), 30% v/v glycerol, and 0.03% w/v bromophenol blue and boiled at 95 °C for 5 min. Total proteins (25 μg) were separated by electrophoresis in 5–20% SDS-polyacrylamide gradient gels and electrophoretically transferred onto polyvinylidene difluoride (PVDF) membranes. After transfer, the membranes were washed in Tris-buffered saline containing 0.1% Tween 20 (TBST) and blocked with 5% skim milk in TBST for 1 h at room temperature. After blocking, membranes were washed and incubated with primary antibodies against phospho-Akt (Ser473, cat# 9271), Akt (cat# 2920), phospho-mTOR (Ser2448, cat# 2971), mTOR (cat# 4517), phospho-p70S6K (Thr389, cat# 9205), p70S6K (cat# 9202), phospho-rpS6 (Ser240/244, cat# 2215), rpS6 (cat# 2317), phospho-ERK1/2 (Thr202/Tyr204, cat#4370), ERK1/2 (cat# 4696), phospho-AMPK (cat# 2531), AMPK (cat# 2532), phosphor-ULK1 (Ser317, cat# 12753, Thr757, cat#14202), ULK1 (cat# 8054), LC3 (cat# 2775), polyubiquitin (cat# 3936) (Cell Signaling Technology, Danvers, MA, USA), and p62 (cat# MP045) (Medical & Biological Laboratories, Aichi, Japan). Membranes were washed in TBST, then incubated with appropriate secondary antibodies. Protein bands were detected by chemiluminescence (GE Healthcare, Harrisburg, PA, USA, or Merck Millipore, Darmstadt, Germany) and analyzed by densitometry using a chemiluminescence detector (ImageQuant LAS 4000; GE Healthcare). BlotsThe membranes were subsequently stained with Coomassie Blue to verify equal loading in all lanes. Band intensities were quantified using ImageJ version 1.46 (National Institutes of Health, Bethesda, MD, USA).

#### Real-time polymerase chain reaction (PCR)

Total RNA was extracted from each powdered muscle sample using ISOGEN I (Nippon Gene, Tokyo, Japan) according to the manufacturer’s instructions. Total RNA concentrations were measured using a NanoDrop 2000 spectrophotometer (Thermo Fisher Scientific), and 500 ng of total RNA was reverse transcribed into cDNA using the PrimeScript™ RT Master Mix (Takara Bio, Shiga, Japan). The cDNA product was mixed with TaqMan Master Mix, primers, TaqMan probes, and RNase- and DNase-free water and analyzed on an ABI 7500 Fast Real-Time PCR System (Applied Biosystems, Foster City, CA, USA). Primers and probes were designed to specifically amplify the sequences of rat MAFbx/atrogin-1, MuRF1, and glyceraldehyde-3-phosphate dehydrogenase (*GAPDH*) (GenBank accession numbers: MAFbx/atrogin-1, NM_133521.1; MuRF-1, NM_080903.1; GAPDH, NM_017008.3). The housekeeping gene *GAPDH* was used as an internal control, and the relative quantification of gene expression was performed using the comparative threshold cycle ΔΔCT method.

#### Muscle protein synthesis

Muscle protein synthesis was measured using the in vivo surface sensing of translation (SUnSET) method as described previously [30–32]. Briefly, 0.04 mmol puromycin/g body weight (MilliporeSigma, Burlington, MA, USA) diluted using a 0.02 mol/L PBS stock solution was intraperitoneally injected after 5 min of anesthesia, and muscle was removed exactly 15 min after puromycin administration. Following homogenization and centrifugation at 2000×g for 3 min at 4 °C, the supernatant was collected and processed for western blotting. A mouse monoclonal antipuromycin antibody (cat# MABE343) (MilliporeSigma) was used to detect puromycin incorporation, which was calculated as the sum of the intensity of all protein bands in the western blot.

#### Statistical analyses

Student’s *t* test was used to evaluate the group differences of food consumption, body weight and wet weight of left gastrocnemius muscle between NOR and AME group. Two-way analysis of variance (ANOVA) (diet × resistance exercise) was used to evaluate changes in protein phosphorylation and gene expression. Post hoc analyses were performed using the Tukey-Kramer test when significant interaction was found. All values are expressed as means ± standard error of the mean (SEM). The level of significance was set at *P* < 0.05.

## Results

### Food consumption and body weight

There was no significant difference in the amount of food consumption nor body weight between the NOR and AME groups throughout the entire experimental period (Table 2).

**Table 2:** Food consumption and body weight

	NOR			AME		
	Day 2	Day 4	Day 7	Day 2	Day 4	Day 7
Food consumption (g)	24.4 ± 1.0	28.3 ± 0.8	28.0 ± 0.9	25.0 ± 1.5	24.9 ± 1.3	28.2 ± 0.7
Body weight (g)	436.8 ± 5.5	450.2 ± 2.9	468.3 ± 4.5	446.1 ± 8.5	447.1 ± 5.1	463.8 ± 6.4

Values are mean ± SEM. *NOR* Rats fed normal chow, *AME* Rats fed chow containing *Aronia melanocarpa* extract

#### Muscle wet weight

To evaluate the effect of 1 week of AME supplementation without exercise on muscle mass, we measured the wet weight of the control, unexercised left gastrocnemius muscle, because a previous study has observed that chronic UA supplementation without exercise was sufficient to skeletal muscle hypertrophy in mice [14]. The mean wet weights were not statistically different at 2.55 ± 0.03 and 2.49 ± 0.05 g in the NOR and AME groups, respectively.

#### Akt

**Fig. 2:**
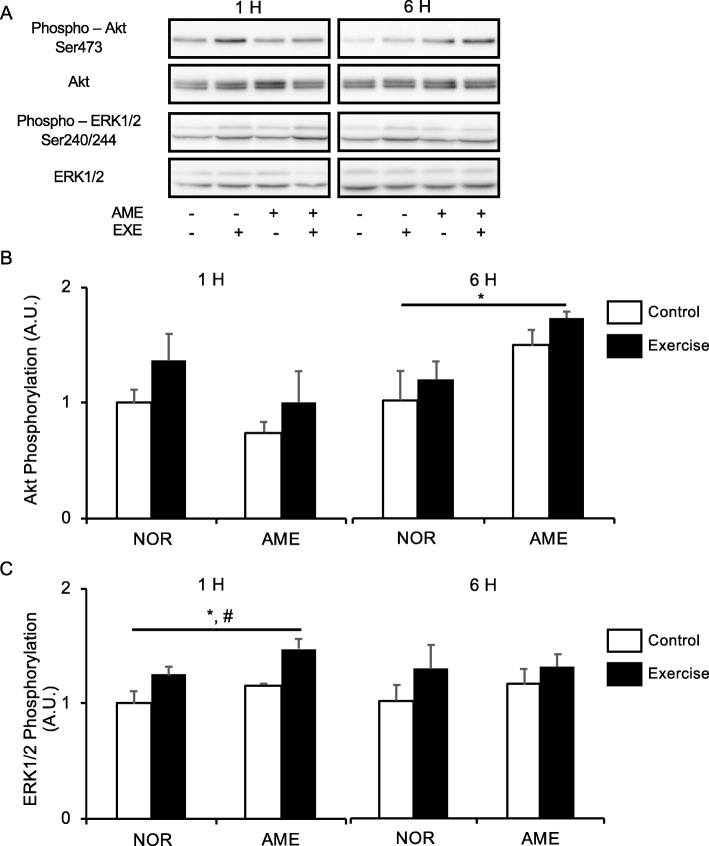
Phosphorylation of upstream substrates of mTORC1 relative to total protein content after resistance exercise. **a** Representative western blots. **b** Phosphorylated Akt at Ser473. **c** Phosphorylated ERK1/2 at Ser240/244. Values are means ± SEM. *P < 0.05, effect of diet; #*P* < 0.05, effect of exercise. AME, *Aronia melanocarpa* extract. EXE, exercise. A.U., arbitrary units

Neither exercise nor diet affected Akt phosphorylation at Ser473 (Fig. 2b) at 1 h after exercise. However, at 6 h after exercise, Akt phosphorylation was higher in the AME group than in the NOR group. Exercise and the interaction of exercise and diet did not affect phosphorylation of Akt at 6 h after exercise.

#### ERK1/2

Both resistance exercise and AME consumption alone significantly increased ERK1/2 phosphorylation at Ser240/244 (Fig. 2c) at 1 h after exercise. No significant differences in the effect of exercise, diet, and interaction thereof were observed at 6 h after exercise.

#### mTOR

**Fig. 3:**
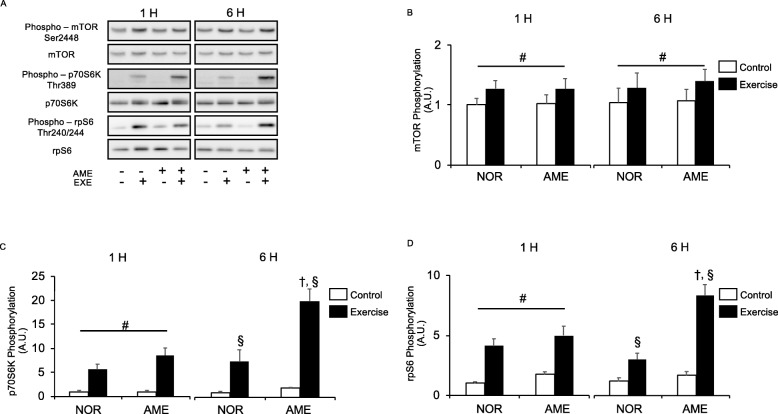
Phosphorylation of markers of mTORC1 activity relative to total protein content after resistance exercise. **a** Representative western blots. **b** Phosphorylated mTOR at Ser2448. **c** Phosphorylated p70S6K at Thr389. **d** Phosphorylated rpS6 at Thr240/244. Values are means ± SEM. #*P* < 0.05, effect of exercise; †*P* < 0.05 vs. control leg in the same group; §*P* < 0.05 vs. corresponding leg in the NOR group. AME, *Aronia melanocarpa extract*. EXE, exercise. A.U., arbitrary units

Resistance exercise significantly increased phosphorylation of mTOR at Ser2448 (Fig. 3b) at 1 and 6 h after exercise. AME supplementation did not increase mTOR phosphorylation at either time point.

#### p70S6K

Neither diet nor the interaction of diet and exercise significantly changed the phosphorylation of p70S6K at Thr389 (Fig. 3c) at 1 h after exercise. Resistance exercise significantly increased the phosphorylation of p70S6K at Thr389 in both the NOR and AME groups at both 1 and 6 h after exercise. Furthermore, AME consumption significantly enhanced the exercise-induced phosphorylation of p70S6K at 6 h.

#### rpS6

Neither diet nor the interaction of diet and exercise changed the phosphorylation of rpS6 at Ser240/244 (Fig. 3d) at 1 after exercise. However, rpS6 phosphorylation in both the NOR and AME groups was significantly increased at 1 and 6 h by exercise. Furthermore, AME consumption further enhanced the exercise-induced phosphorylation of rpS6 at 6 h.

#### Protein synthesis rate

**Fig. 4:**
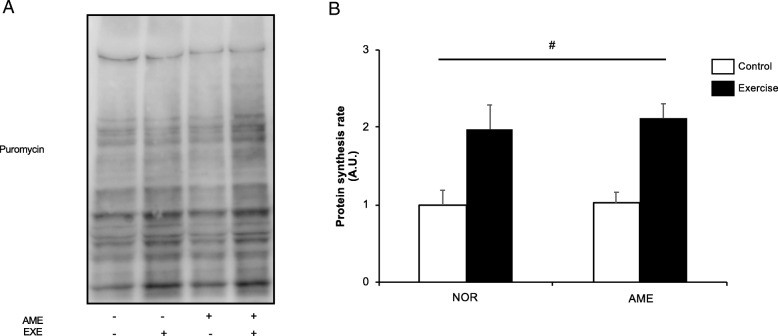
Muscle protein synthesis rate at 6 h after resistance exercise. **a** Representative western blots. **b** Quantification of muscle protein synthesis. Values are means ± SEM. # *P* < 0.05, significant main effect of exercise. A.U., arbitrary units. AME, *Aronia melanocarpa* extract, EXE, exercise

Resistance exercise significantly increased the rate of protein synthesis at 6 h following exercise (Fig. 4b). AME supplementation had no significant effect.

#### AMPK

**Fig. 5:**
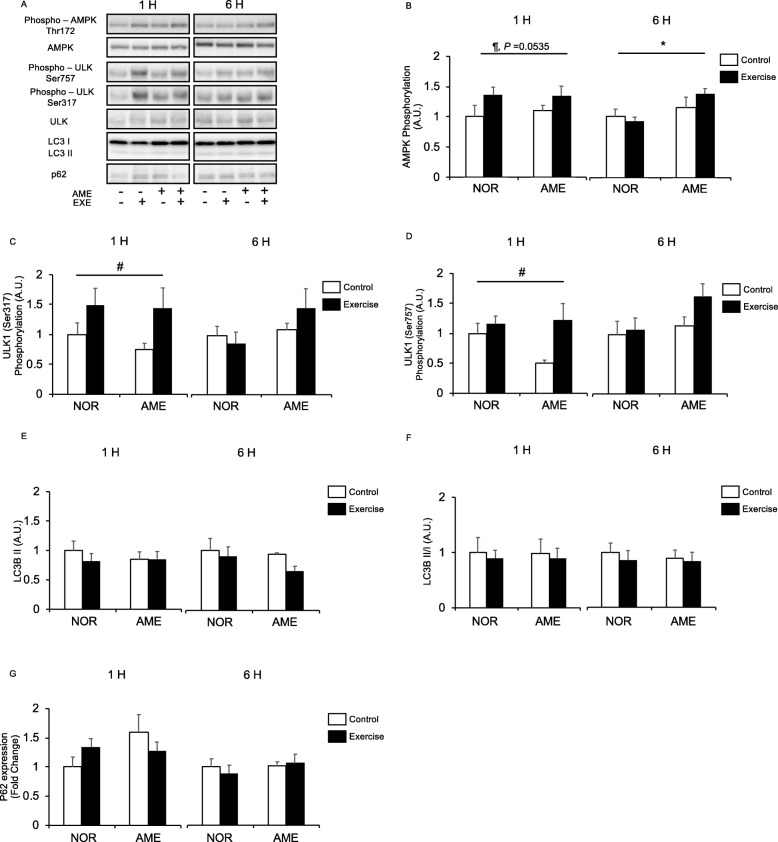
Phosphorylation relative to total protein content and expression of autophagy-lysosome system mediators after exercise. **a** Representative western blots. **b** Phosphorylated AMPK at Thr172. **c** Phosphorylated ULK1 at Thr757. **d** Phosphorylated ULK1 at Ser317. **e** LC3B-II expression. **f** Ratio of LC3B-II to -I. **g** P62 expression. Values are means ± SEM. *P < 0.05, effect of diet; #*P* < 0.05, effect of exercise; ¶ trend for exercise. AME, *Aronia melanocarpa* extract. EXE, exercise. A.U., arbitrary units

AMPK phosphorylation at Thr172 was increased at 1 h after resistance exercise, though not significantly (Fig. 5b). AME supplementation alone had no effect at 1 h after exercise. At 6 h, we observed an increase in AMPK phosphorylation in the AME group, but observed no statistically significant effect from exercise and interaction of diet and exercise.

#### ULK1

ULK1 is phosphorylated at Ser317 and activated by AMPK [33], whereupon it increases autophagosome formation and subsequently promotes protein degradation [34]. ULK1 has a second phosphorylation site at Ser757, which is regulated by mTORC and negatively regulates ULK1 activity [16, 33]. Resistance exercise significantly increased ULK1 phosphorylation at both Ser317 (Fig. 5c) and Ser757 (Fig. 5d) only at the 1 h time point. Diet and interaction of diet and exercise did not affect phosphorylation at either site at either time point.

#### LC3B-ii

LC3B-II, formed by phosphatidylethanolamine conjugation of LC3B-I, binds to autophagosomes [35]. LC3B-II is used as a marker of autophagy because its levels are correlated with numbers of autophagosomes [36]. We observed no significant differences in LC3B-II expression (Fig. 5e) associated with diet or exercise. Furthermore, the ratios of LC3B-II to -I (Fig. 5f) were not significantly different between groups at either time point.

#### P62

Diet, resistance exercise, and interaction thereof did not significantly affect p62 protein expression (Fig. 5g) at both 1 and 6 h after resistance exercise.

#### MAFbx and MuRF1

**Fig. 6:**
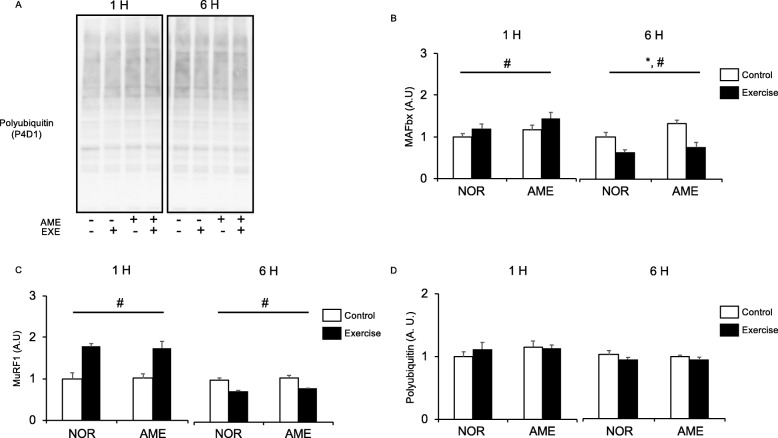
Expression of polyubiquitinated proteins and mRNA expression of mediators of UPS after resistance exercise. **a** Representative western blots. **b** MAFbx expression. **c** MuRF1 expression. **d** Polyubiquitinated protein expression. Values are means ± SEM. *P < 0.05, effect of diet; #*P* < 0.05, effect of exercise. AME, *Aronia melanocarpa* extract. EXE, exercise. A.U., arbitrary units

MAFbx mRNA levels were significantly increased (Fig. 6b) at 1 h by resistance exercise. Neither diet nor the interaction of exercise and diet affected MAFbx mRNA levels at 1 h. At 6 h, we observed an increase in MAFbx mRNA expression in the AME group, but the effect of exercise was no longer present. On the other hand, MuRF1 mRNA expression was significantly increased (Fig. 6c) at 1 h after resistance exercise. Neither diet nor the interaction of exercise and diet affected MuRF1 mRNA expression at 1 h. There was no significant difference in MuRF1 mRNA expression between the NOR and AME groups at 6 h after exercise.

#### Polyubiquitinated protein

Diet, resistance exercise, and interaction thereof did not significantly affect protein polyubiquitination (Fig. 6d) at both 1 and 6 h after resistance exercise.

## Discussion

In this study, we investigated the effects of AME, derived from a fruit rich in ursolic acid, alone and in combination with resistance exercise on phosphorylation of anabolic signaling proteins, MPS, and mediators of protein degradation. We found that AME enhanced mTORC1 activity in response to resistance exercise, but did not affect MPS and muscle protein degradation mediators.

The mean amount of food consumption did not differ between the NOR and AME groups; nor did body weight on the day of the exercise experiment. These results indicate that AME did not alter the feeding habits of the experimental rats. The rats in the AME group consumed 26 g/day of AME chow, resulting in a daily ursolic acid intake of approximately 115 mg/kg body weight. Kukel et al. administered chow containing 0.14% ursolic acid to mice for 5 weeks and demonstrated that the supplementation prevented fasting-induced muscle atrophy and induced muscle hypertrophy in the absence of exercise [14]. Considering that mice consume approximately 3 g chow per day, we inferred that these mice received 140 mg/kg body weight of ursolic acid daily. In the present study, we found that AME supplementation alone did not induce hypertrophy, as the weight of unexercised control gastrocnemius muscles did not differ between NOR and AME groups. This was not consistent with the data of Kukel et al., as the amount of ursolic acid ingested in our study was almost the same. The discrepancy between these results may be attributable to the difference in the duration of supplementation (1 week vs. 5 weeks) or to compounds in AME other than ursolic acid. Thus, longer durations of AME supplementation may be required to increase muscle mass without exercise in humans.

Akt and ERK1/2 are upstream substrates involved in regulation of mTORC1 activity [37–39]. AME increased phosphorylation of both of them in this study, consistent with a previous report using ursolic acid [14]. Moreover, *A. melanocarpa* juice, rich in ursolic acid, stimulated Akt phosphorylation in endothelial cells [40]. To our knowledge, this study is the first to show that AME enhances Akt and ERK1/2 phosphorylation in skeletal muscle. However, the phosphorylation levels of p70S6K and rpS6 did not completely reflect those of Akt and ERK1/2. Further study is needed to clarify the molecular mechanisms underlying enhancement of mTORC1 activation in response to resistance exercise by AME supplementation.

We found that resistance exercise increased the phosphorylation of p70S6K and rpS6. Furthermore, phosphorylation was increased in the AME group at 6 h after exercise, suggesting synergy between AME and exercise, in agreement with a previous study that observed augmentation of resistance exercise-induced mTORC1 activation by ursolic acid at 6 h following resistance exercise [15]. Thus, it was suggested that the positive effects of AME on mTORC1 activation after resistance exercise may be due to the ursolic acid in AME. Interestingly, although the prior study showed that ursolic acid exerted no effect on the phosphorylation of rpS6, a downstream substrate of p70S6K, in response to resistance exercise [15], we demonstrated that AME enhanced phosphorylation of rpS6 as well as p70S6K after exercise. These data show that AME supplementation might achieve better enhancement of mTORC1 activity than ursolic acid supplementation can because AME augmented the phosphorylation of not only p70S6K but also of its downstream substrate. Of note, ursolic acid was injected as a single dose before exercise in the previous study [15], whereas our rats were administered food containing AME for 1 week. This suggests that longer-term supplementation with AME may be more effective for activation of mTORC1 and increasing muscle mass. This is consistent with the work cited above, which showed that 5 weeks of UA supplementation induced muscle hypertrophy without exercise [14].

Our data showed that AME enhanced exercise-induced p70S6K phosphorylation, a marker of mTORC1 activity; nevertheless, AME did not enhance MPS after exercise. We have previously demonstrated that rapamycin inhibited mTORC1 activation, but partly decreased MPS increase after muscle contraction [6]. Moreover, You et al. have shown that conditional knockout of Raptor, an important component of mTORC1, eliminated mTORC1 activation but did not attenuate increased MPS in response to mechanical load [25]. Thus, our data are in agreement with recent studies showing that mTORC1 activity does not necessarily coincide with increased MPS after mechanical load.

It has been proposed that chronic resistance training-induced muscle hypertrophy is produced by increases in MPS through mTORC activation after acute exercise [41, 42]. However, a recent study demonstrated that mTORC1 activation, but not increased MPS, is necessary for muscle hypertrophy induced by mechanical load [25]. Therefore, mTORC1 may be a better predictor of muscle hypertrophy than acute increases in MPS after muscle contraction. Consistent with this hypothesis, Mitchell et al. have shown that increased p70S6K phosphorylation, but not MPS, is correlated with resistance training-induced muscle hypertrophy [5, 43]. Hence, although we could not observe the enhancement of MPS in the AME group in the present study, AME supplementation with chronic resistance training could accelerate muscle hypertrophy by enhancing mTORC1 activation. On the other hand, a previous study reported that the combination of ursolic acid supplementation and resistance training did not induce skeletal muscle hypertrophy in humans [44]. As mentioned above, our present data suggested that AME, which is not only rich in ursolic acid but also various other compounds, could achieve better enhancement of mTORC1 activity compared with ursolic acid alone. Thus, AME supplementation might more effectively accelerate muscle hypertrophy after chronic resistance training than ursolic acid alone. To test this hypothesis, chronic studies in humans are needed to better understand the effects of AME in conjunction with resistance training. Moreover, it should be considered that nutritional status is involved in controlling mTORC1 activity. In this study, the effect of AME was investigated in a fasted state. However, a previous study on human subjects demonstrated that nutritional status enhanced mTORC1 activation after resistance exercise [21]. Thus, we need to clarify whether the combination of AME and other anabolic nutritional intake is effective in future experiments. These data may lead to improved exercise and nutritional strategies.

Activation of AMPK, which upregulates autophagy and UPS-related signaling, is an important marker of muscle protein degradation. We observed that AME supplementation increased AMPK Thr172 phosphorylation. A previous study reported that ursolic acid treatment increased phosphorylation of AMPK at the same residue in C2C12 myotubes [45], suggesting that ursolic acid could activate intramuscular AMPK. Indeed, another study demonstrated that ursolic acid supplementation activated AMPK in obese rats [46]. Thus, the increased AMPK phosphorylation induced by AME supplementation is likely produced by the ursolic acid in AME. However, the other components of AME, such as anthocyanin, chlorogenic acid, and protocatechuic acid, on AMPK phosphorylation should be isolated and tested because prior research has demonstrated that these compounds stimulate AMPK activity [47–49].

As in our previous studies, we observed that exercise increased ULK1 phosphorylation at both Ser317 and Ser757 in this study [50]. However, AME, both alone and in combination with resistance exercise, did not affect ULK1 phosphorylation. Furthermore, the levels of LC3B-II expression, a marker of autophagy, and the ratio of LC3B-II to LC3B-I were unchanged by AME. Additionally, AME alone and in combination with resistance exercise failed to alter expression of the autophagic flux marker p62. Collectively, these data suggest that AME supplementation has no effect on the autophagy-lysosome protein degradation system.

MAFbx and MuRF1 are muscle-specific ubiquitin ligases. We observed that AME supplementation increased both AMPK phosphorylation and MAFbx expression at 6 h after resistance exercise. A previous study showed that AMPK activation increased the expression of ubiquitin ligases in C2C12 cells [8]. In agreement with those results, our data showed that MAFbx expression increased concomitantly with the upregulation of AMPK phosphorylation. However, the results of the earlier study showed that ursolic acid consumption for 5 weeks decreased ubiquitin ligase expression, which appears to be inconsistent with our data [14]. The discrepancy may be the result of differences in supplement composition, i.e., ursolic acid alone or in combinations. Alternatively, the effects of ursolic acid and/or AME on ubiquitin ligase might differ depending on physiological conditions, such as the resting vs. recovery from exercise. Another possible reason for the difference is that the treatment periods were different. Longer supplementation duration may be needed to decrease MAFbx expression. While both resistance exercise and AME supplementation altered ubiquitin ligase expression, neither changed polyubiquitin levels. These results suggest that both resistance exercise and AME affect gene expression involved in UPS but have little effect on the physiological response.

Notably, the effect of AME on MAFbx was observed only at 6 h after exercise. AME supplementation also altered several other parameters only at the 6-h time point. Furthermore, ursolic acid has been shown to enhance resistance exercise-induced mTORC1 activation at the same time point [15]. Although it is difficult to explain why AME affected signaling factors only at 6 h and not at 1 h, AME/ursolic acid might affect the middle phase of the physiological response to resistance exercise. Moreover, it should be noted that it was uncertain whether AME actually affected signaling substrates at 6 h after resistance exercise. A previous study using human subjects found a discordance between increase in mTORC1 activity and MPS after oral protein supplementation [51]. Thus, in our study, AME may have increased MPS at times other than 6 h after resistance exercise. Similarly, AME may have affected other signaling substrates at the different time points. Taken together, having only two time points is a limitation of this study.

## Conclusion

AME, which is rich in ursolic acid, enhanced mTORC1 activation in response to resistance exercise. On the other hand, AME did not affect MPS and accelerate muscle protein degradation or otherwise have a negative effect on protein metabolism. As mTORC1 activation after resistance exercise is necessary for muscle hypertrophy, our present data showed the potential of AME for enhancing muscle hypertrophy induced by chronic resistance training. However, to establish practical nutritional strategies involving AME, further studies are needed to clarify how AME enhances mTORC1 activity and the effect of the combination of AME and chronic resistance exercise on muscle hypertrophy in humans.

## Data Availability

All data generated or analyzed during this study are included in this published article.

## Abbreviations

AME: *Aronia melanocarpa* extract
AMPK: AMP-activated protein kinase
MAFbx: Muscle atrophy F box
MPS: Muscle protein synthesis
mTORC1: mechanistic target of rapamycin complex 1
MuRF1: Muscle-specific RING finger 1
NOR: Normal chow
p70S6K: p70S6 kinase
rpS6: ribosomal protein S6
SDS: Sodium dodecyl sulfate
ULK1: UNC-51 like kinase
UPS: Ubiquitin-proteasome system
